# Calcifying Odontogenic Cyst with Extensive Areas of Dentinoid: Uncommon Case Report and Update of Main Findings

**DOI:** 10.1155/2018/8323215

**Published:** 2018-05-10

**Authors:** Hellen Bandeira de Pontes Santos, Everton Freitas de Morais, Deborah Gondim Lambert Moreira, Luis Ferreira de Almeida Neto, Petrus Pereira Gomes, Roseana de Almeida Freitas

**Affiliations:** ^1^Postgraduate Program in Oral Pathology, Federal University of Rio Grande do Norte, Natal, RN, Brazil; ^2^Oral and Maxillofacial Surgery Residence, Federal University of Rio Grande do Norte, Natal, RN, Brazil

## Abstract

The calcifying odontogenic cyst (COC) is a benign odontogenic cyst that occurs in the gnathic bones. This cyst is part of a spectrum of lesions characterized by odontogenic epithelium containing “ghost cells,” which may undergo calcification. Areas of an eosinophilic matrix material compatible dentinoid also may present adjacent to the epithelial component. However, these areas of dentinoid commonly do not appear so abundant in COCs. In this study, we report a case of intraosseous COC with extensive areas of dentinoid and perform an update regarding the clinical, radiographical, histopathological, and differential diagnosis, treatment, and prognosis of this cystic lesion.

## 1. Introduction

The calcifying odontogenic cyst (COC) was first described by Gorlin and colleagues in 1962 [1], which described a cyst lined by an ameloblastoma-like epithelium that contained variable amounts of ghost cells and calcifications. This cyst is not common in the gnathic bones, representing about less than 6% of all odontogenic lesions [2, 3].

Recently, this lesion was reclassified as a cystic lesion again by the World Health Organization (WHO) [4]. It was showed in an international collaborative study that just under 90% of these lesions are either entirely cystic or associated with odontomas [5]. For this reason, the WHO's group believe that there is no justification for classifying these lesions as neoplastic [6].

COCs represent a heterogeneous group of lesions that show a variety of clinicopathologic and behavioral features [7]. Intraosseus COCs commonly occur with similar frequency both gnathic bones and majority of cases are found in the incisor and canine areas [8–10]. Radiographically, these lesions are usually an unilocular, well-defined radiolucency, although the lesion occasionally may appear multilocular. Radiopaque structures within the lesion, either irregular calcifications or tooth-like densities, may also be present in some cases [8–11].

The distinct feature of COC is a cystic lining demonstrating “ghost” epithelial cells with a propensity to calcify. This cyst most commonly occurs as a well-defined cystic lesion with a fibrous capsule and a lining of odontogenic epithelium with Ameloblastomatous appearance [10, 11]. Areas of an eosinophilic matrix material that are considered by some authors to represent dentinoid also may be present adjacent to the epithelial component. This is believed to be the result of an inductive effect by the odontogenic epithelium on the adjacent mesenchymal tissue [10, 11]. However, to the best of our knowledge, these areas of dentinoid commonly do not appear so abundant in COCs. Herewith, we report a case of intraosseous COC with extensive areas of dentinoid and perform an update regarding the clinical, radiographical, histopathological, and differential diagnosis, treatment, and prognosis of this cystic lesion.

## 2. Case Report

An 82-year-old male patient presented in the Oral and Maxillofacial Surgery Service of the Federal University of Rio Grande do Norte for evaluation of a swelling on the alveolar ridge of the anterior mandible, which had been previously identified in routine radiographic examination with prosthesis purposes. During anamnesis, the patient reported to be hypertensive and diabetic, as well as having chronic heart disease. At the clinical examination, no volume could be observed in the anterior region of the mandible, and the patient reported no painful symptomatology.

**Figure 1:**
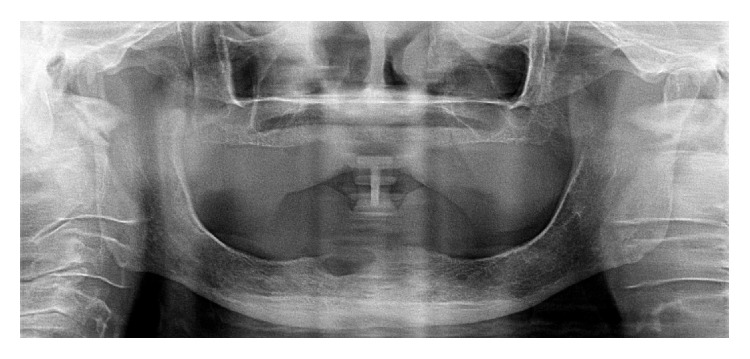
Panoramic radiography showing an unilocular radiolucent lesion in anterior mandible.

**Figure 2:**
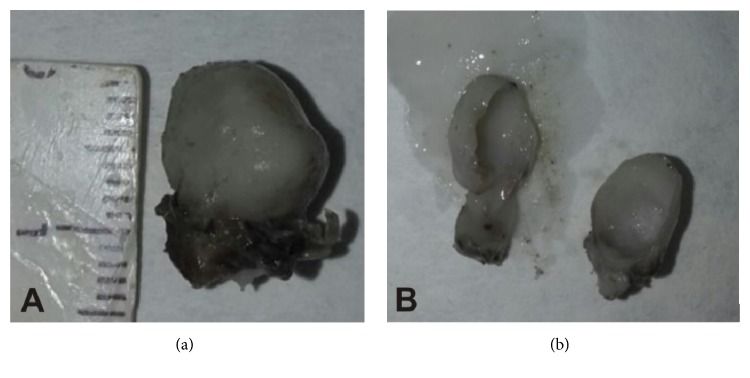
Gross aspect revealing a firm and oval mass with cystic aspect (a), containing liquid in the interior (b).

**Figure 3:**
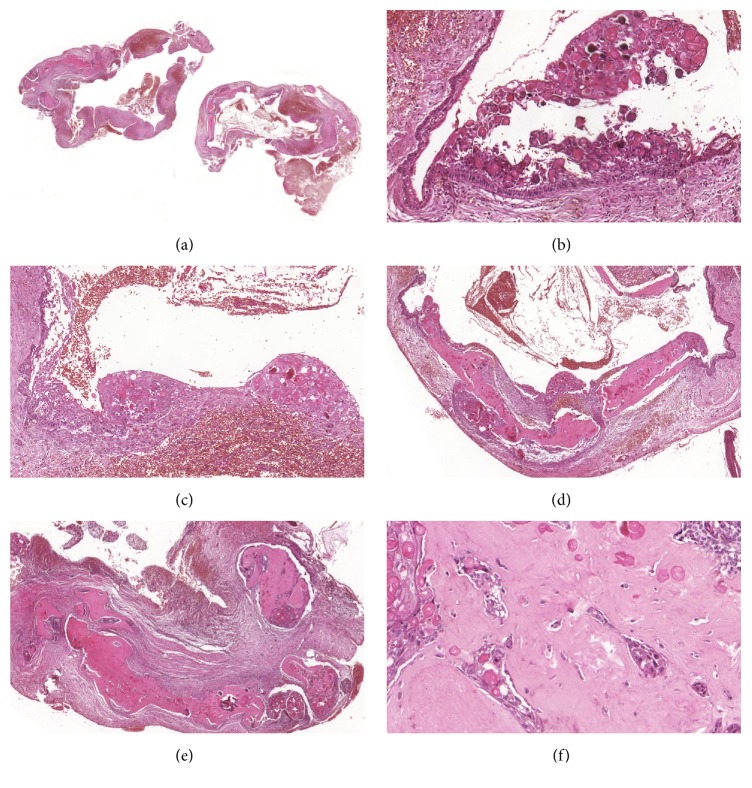
(a) Photomicrography showing a cystic lesion with a fibrous capsule and a lining of odontogenic epithelium (H/E, 40x). (b-c) Highlight for the columnar basal cells of similar to ameloblasts and the overlying layers loosely arranged with several amounts of ghost cells (H/E, 200x). (d–f) Extensive areas of eosinophilic matrix compatible with dentinoid within the fibrous capsule adjacent to odontogenic epithelium (H/E, 200x, 400x).

**Figure 4:**
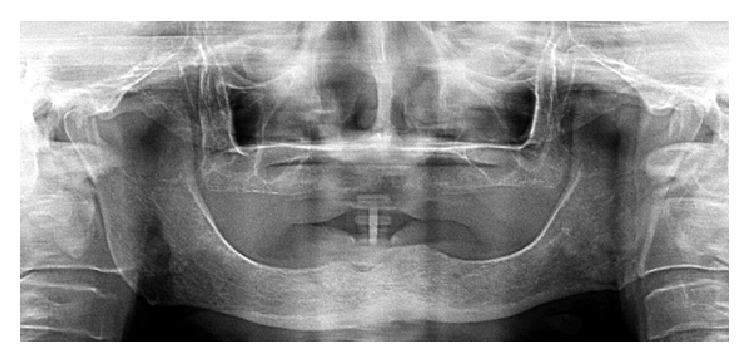
Panoramic radiography revealing bone neoformation after 6 months of the surgical procedure.

Radiographical examination revealed an unilocular and irregular radiolucent lesion, measuring approximately 3 cm in diameter (Figure 1). Preoperative laboratory tests (hemogram, coagulogram, and blood glucose testing) were performed, as well as surgical risk and preanesthetic evaluation. Under the diagnostic hypothesis of residual cyst or another odontogenic lesion, the patient underwent surgery under general anesthesia in order to enucleate the lesion. In the transoperative period, in addition to surgical enucleation, a peripheral osteotomy and interposition of synthetic material for guided bone regeneration (Bio-OSS and Bio-Gide) were performed, considering the need for prosthetic rehabilitation of the patient. After the procedure, the specimen was sent to histopathological examination. Gross examination of the specimen revealed a firm and oval mass with cystic aspect, containing liquid in the interior (Figures 2(a) and 2(b)). Microscopically, the hematoxylin and eosin (H and E) stained section showed a defined cystic lesion with a fibrous capsule and a lining of odontogenic epithelium (Figures 3(a)–3(c)). The basal cells of the epithelial lining were mainly columnar and similar to ameloblasts and the overlying layers were loosely arranged, resembling the stellate reticulum of the enamel organ. Several amounts of ghost cells were found within the epithelial component and also in the capsule (Figure 3(c)). Extensive areas of eosinophilic matrix compatible with dysplastic dentin (dentinoid) were found in the fibrous capsule (Figures 3(d)–3(f)). After six months, bone neoformation has been observed and there has been no clinical or radiographic evidence of recurrence (Figure 4). The patient remains under follow-up.

## 3. Discussion

The COC is a rare entity of uncertain pathogenesis; its clinical and radiographic characteristics are not pathognomonic, being characterized mainly by its histopathological characteristics [12]. This cystic lesion is generally asymptomatic, and its radiographic features usually include an unilocular radiolucent lesion with focal areas of radiopacity [13]. However, it can occur also in extraosseous regions, as in the gingiva, corresponding to 15 to 25% of all reported cases of COC [4, 12, 13].

Since its recognition and description by Gorlin et al. (1962) [1], the biological behavior of the COC is still a subject of much debate. According to the current classification of odontogenic tumors by the WHO, this lesion, which was previously known as calcifying cystic odontogenic tumor, has now returned to be considered a cystic lesion [4].

Approximately 65% of all cases of COC are reported in the anterior region of the mandible [14]. This study presents a case diagnosed in a 70-year-male patient that had a localized lesion in the anterior region of the mandible, similar features to the most frequent clinical and radiographic findings in the literature. COC may be associated with other odontogenic lesions, especially to odontomas (ranging from 22% to 47%), ameloblastomas, adenomatoid odontogenic tumors, and ameloblastic fibromas [15]. In our study, there was no association of the cyst with another odontogenic lesion.

The histopathological findings of the COC include basal layer of the epithelial lining presenting a columnar or cuboid appearance with a similar appearance to ameloblasts. It is also possible to observe a cellular arrangement that resembles the stellate reticulum of the enamel organ in the suprabasal layers [9, 14]. A significant feature of this lesion is the presence of anucleated and slightly eosinophilic cells, which are denominated “ghost cells.” Displasic dentin also is present in the fibrous capsule [10, 13].

In the present case, it was possible to observe a prominent and extensive presence of dentinoid in the fibrous capsule. There are different theories regarding the etiology of this material; while some authors believe that it may be a result of an inductive effect by the odontogenic epithelium on the adjacent mesenchymal tissue [10, 11], others suggest it may be originated from ghost cells [10, 13]. Fregnani et al. [16] also found some lesions with extensive areas of dentinoid in a histological and immunohistochemical evaluation of ten cases of COCs. This characteristic was observed in three cysts; two of them were associated with odontomas and the other was an extraosseous variant. In all the three cases, the representative dentinoid was located near the epithelium [16], what differs from the present study, in which the dentinoid extended from near the epithelium to deeper areas of the fibrous capsule. Furthermore, the literature shows that these areas of dentinoid commonly do not appear so extensive in COCs, as seen in the current case.

Intraosseous COC is mainly treated by enucleation, with a low rate of recurrence, occurring in <5% of cases [8]. However, if this tumor is associated with another odontogenic tumor, the treatment and prognosis are likely to be same as for the associated tumor. There are reports of malignant transformation in recurrent cases of COC. Thus, long-term follow-up of such patients should be performed [17, 18].

In conclusion, the present case reinforces the importance of knowledge by the pathologists of the histopathological features of the intraosseous COC in order to achieve a correct diagnosis and, consequently, a favorable outcome for the patient. Furthermore, the literature shows that these areas of dentinoid commonly do not appear so extensive in COCs, as seen in the current case.
